# Implantation of a Newly Designed Supratarsal Gold Weight versus the Traditional Pretarsal Model for the Correction of Long-standing Paralytic Lagophthalmos: A Retrospective Cohort Study

**DOI:** 10.1055/s-0043-1777287

**Published:** 2024-02-29

**Authors:** Natthiya Lailaksiri, Pawarit Wanichsetakul, Preamjit Saonanon

**Affiliations:** 1 Department of Ophthalmology, Faculty of Medicine, Chulalongkorn University, King Chulalongkorn Memorial Hospital, Bangkok, Thailand; 2 Department of Ophthalmology, Faculty of Medicine, Thammasat University, Bangkok, Thailand

**Keywords:** lagophthalmos, facial palsy, lid load, gold weight implantation, supratarsal implantation

## Abstract

**Background** The study determined to compare the clinical outcomes of traditional gold weight implantation for the correction of paralytic lagophthalmos with those of a newly designed model.

**Methods** In this retrospective cohort study, we enrolled 30 patients (76% females; average age 60.8 ± 12 years) with facial palsy who underwent implantation of either the traditional pretarsal gold weight (PT group; *n* = 15) or a new supratarsal model (ST group; *n* = 15) from May 2014 to April 2019. The main outcome measures were the 12-month postoperative weight prominence, weight migration, improvement of lagophthalmos, upper eyelid contour, and upper eyelid ptosis. The secondary outcome was long-term (24 months) reoperative rate.

**Results** The new model group had significantly better eyelid contour (risk ratio [RR] 3.16, 95% confidence interval [CI] 1.62–6.15, *p* = 0.001), less weight prominence (RR 1.74, 95% CI 1.13–2.70, *p* = 0.013), less weight migration (RR 1.31, 95% CI 1.12–1.54, *p* = 0.001), and less eyelid ptosis (RR 2.36, 95% CI 1.21–4.59, *p* = 0.011) than the traditional model group. Improvement of lagophthalmos was not statistically significant between the two groups (RR 1.44, 95% CI 0.72–2.91, *p* = 0.303). The 24-month reoperative rate was 53.3% in the PT group versus 13.3% in the ST group (RR 2.00, 95% CI 1.15–3.49, *p* = 0.015).

**Conclusion** The newly designed supratarsal gold weight showed superior postoperative outcomes than the standard traditional model.

## Introduction

Paralytic lagophthalmos in lower motor neuron facial nerve palsy can lead to exposure keratitis, which can progress to severe ocular infection and corneal scarring. Current permanent surgical treatment modalities for paralytic lagophthalmos include static and dynamic options. Reanimation of the upper face is a dynamic option preferred only in patients with paralysis for less than 24 months. For long-standing cases, static treatment modalities, including permanent tarsorrhaphy, upper lid loading surgery, and levator lengthening procedures, are more favorable. 1 2 Upper lid loading surgery is a procedure of choice owing to its effectiveness, simplicity, and reversibility. However, the occurrence of postoperative, long-term, implant-related complications is fairly common and usually results in revision surgeries. 3 4

The traditional gold weight model is designed to be placed anterior to the tarsus (pretarsal area) of a Caucasian eye with a trapezoid tarsal shape. 5 Visibility of the edge of the implant is frequently observed in Asian eyes with a sickle-shaped tarsus, leading to poor cosmetic outcomes and high rates of implant infection, exposure, and reoperation. 6 7 Recently, supratarsal implant placement has become more popular among surgeons due to the reduced implant visibility and exposure rate associated with the procedure. 8 9 10 We redesigned the lid load implant and made it thinner to fit the post-levator space and sickle-shaped tarsus of Asian eyes. The primary objective of this study was to compare the clinical outcomes of the newly designed supratarsal gold weight implantation with those of the traditional pretarsal gold weight implantation for the correction of paralytic lagophthalmos in Asian eyes.

## Methods

This was a retrospective cohort study of patients with facial palsy who underwent gold weight implantation performed by a single surgeon (P.S.), from May 2014 to April 2019. We obtained approval for this study from the Institutional Review Board and Ethics Committee, Faculty of Medicine, Chulalongkorn University (IRB No.672/62). This study adhered to the tenets of the Declaration of Helsinki. Data collected included patient demographics and preoperative and postoperative clinical measurements such as upper eyelid contour, upper eyelid ptosis, weight prominence, weight migration, and improvement of lagophthalmos. The 12-month postoperative outcome was graded by five independent, masked, oculoplastic surgeons who assessed subject-standardized photographs. Early and late (24-month) postoperative complications, including exposure keratitis, wound infection, hemorrhage, implant exposure, and reoperative rate, were also recorded. Patients who underwent the surgery but were lost to follow-up were excluded from this study.

**Fig. 1:**
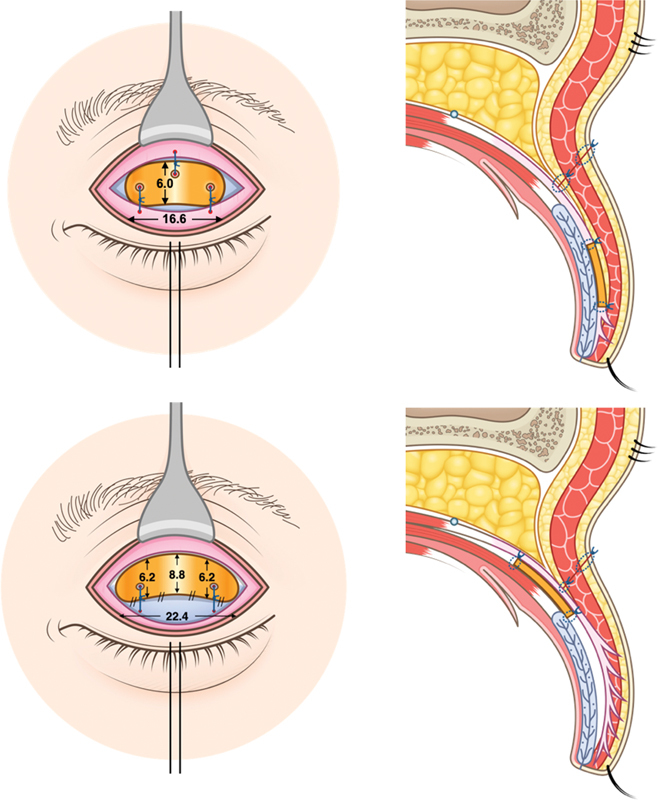
The approximate height and width of the 1.2 g traditional pretarsal gold weight (upper) compared with the newly designed elliptical supratarsal gold weight (lower; courtesy of Wittaya Gasamwattana, 2020, Bangkok: Learnery Co., Ltd, all rights reserved).

The supratarsal gold weight model was designed based on the average dimension minus 2 standard deviations (mean − 2 SDs) of the anatomy of 50 eyelids from 25 Asian cadavers with a sickle-shaped tarsus. The gold weight has a semielliptical shape and a height of 8.8 mm, with the widest base length being 22.4 mm and radius of curvature of 12.7 mm ( Fig. 1 , courtesy of Wittaya Gasamwattana, 2020, Bangkok: Learnery Co., Ltd, all rights reserved).

The weight was created in eight sizes ranging from 0.8 to 2.2 g, with a 0.2 g increase in weight per increase in size. The weight chosen for the traditional pretarsal model is based on the trial weight used in the Tantalum Weight Sizing Set (MedDev, USA), whereas that chosen for the supratarsal model is based on the trial weight of the set plus 0.2 g as recommended by a previous article on supratarsal placement method. 10 The surgical techniques for supratarsal gold weight implantation have been described previously by Caesar et al 8 and were modified as described. Local anesthetic with 2% xylocaine with 1:200,000 adrenaline was done. An eyelid crease incision and dissection to expose the superior tarsal border was performed. The orbital septum is opened and the levator aponeurosis is separated from the Muller's muscle. The gold weight is centered at the superior border of the tarsus and sutured through the medial and lateral holes with two 6/0 polyglycolic acid (PGA) sutures. The edge of the levator aponeurosis was reattached to the tarsus with 6/0 PGA suture in horizontal mattress pattern. Orbicularis and skin are closed with 6/0 PGA and 7/0 nylon suture in an interrupted pattern, respectively.

In the cases of revision surgery, the preexisting gold weight was removed and replaced with the supratarsal model, which was chosen based on the patient's previous implant weight plus 0.2 g. The surgery was performed under local anesthesia as described below. A skin incision was made at the level of the lid crease, 2.5 to 3 cm in length, and monopolar cautery was used to dissect and identify the fibrous capsule around the previous implant. The part of the capsule anterior to the implant was excised and removed. The fixation suture was severed, and the implant was then removed. The part of the capsule posterior to the implant was dissected from the underlying tarsus to the post-levator space. The levator aponeurosis was separated from Muller's muscle to form a single sheath comprising the fibrous capsule and the levator aponeurosis. The gold weight was centered at the upper border of the bare tarsal surface and sutured directly to the upper border of the tarsus with 6/0 PGA sutures and two-point fixation. The fibrous capsule–levator aponeurosis complex was reattached to the upper border of the tarsus, medial and lateral to the implant, using a 6/0 PGA suture in a horizontal mattress pattern. The margin to reflex distance was used to check the eyelid position intraoperatively to prevent unintentional levator advancement. With the eyelid in a proper position, the redundant fibrous capsule was then trimmed. Eyelid crease fixation was performed using 6/0 PGA sutures in an interrupted pattern, from the margin of the fibrous capsule to the pretarsal orbicularis oculi. The skin was closed with a 7/0 nylon suture in an interrupted pattern. Postoperative medication included dicloxacillin (500 mg) four times daily and Maxitrol® eye ointment (Alcon Pharmaceutical, USA) twice daily. The skin sutures were removed 7 to 10 days postoperatively.

**Fig. 2:**
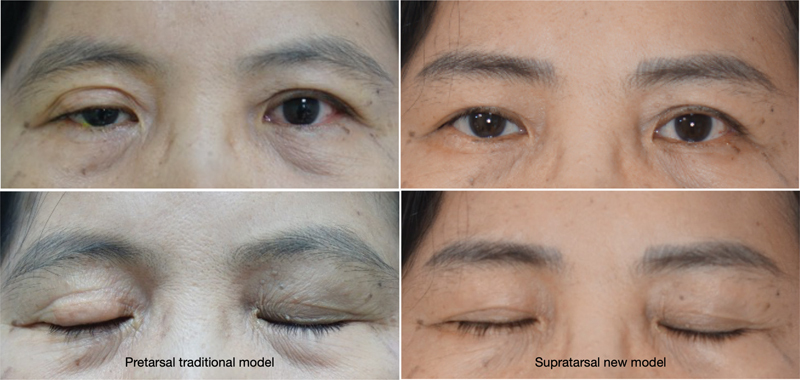
A female patient with complete right facial palsy and history of exposure keratitis and bacterial corneal ulcer of the right eye. She underwent pretarsal implantation of the traditional gold weight 3 years prior and experienced progressive implant exposure and ptosis. The right photos show the 24-month result after implant exchange to the supratarsal gold weight model.

**Fig. 3:**
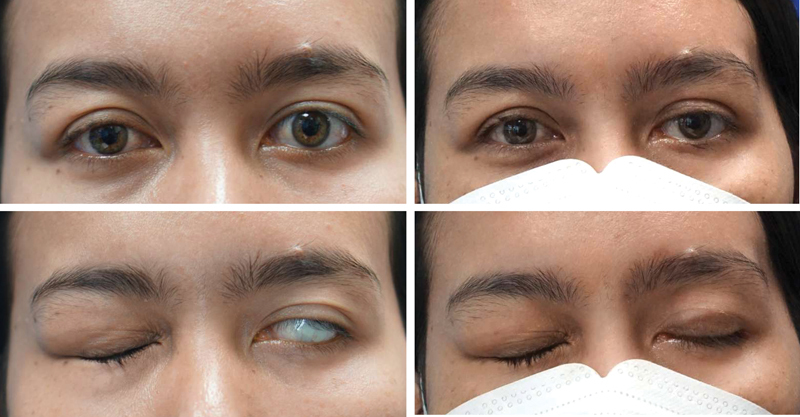
A female patient with complete left facial palsy for 2 years. The right photos show the 24-month result after the primary implant of supratarsal gold weight model.

Five masked oculoplastic surgeons independently graded standard photographs ( Figs. 2 and 3 ) for weight prominence, weight migration, improvement of lagophthalmos, upper eyelid contour, and upper eyelid ptosis. Prior to the assessment of the photographs, the reviewers were informed of the photograph grading system previously reported by Bladen et al. 4 Weight prominence was assessed in eyelid closure photographs and defined as weight visibility on the anterior eyelid (graded as not visible, mild visibility, moderate visibility, or severe visibility). Weight migration was defined as the deviation of the weight from its intended location (graded as yes or no). Upper eyelid contour was scored based on eyelid position, eyelid crease, and eyelid symmetry (graded as good, fair, or poor). Upper eyelid ptosis (graded as clinically significant or not clinically significant) was assessed in photographs in which the patient's eyes were open. Improvement of lagophthalmos was assessed in eyelid closure photographs and defined as improvement of the inability to close the eyelid (graded as no, partial, or complete).

Descriptive statistics were used to evaluate the baseline characteristics of the patients. Categorical data were described as frequencies and percentages. Continuous data with a normal or approximately normal distribution were described using means and standard deviations. Continuous data with skewed distributions were described using medians and interquartile ranges. Comparisons of the primary outcomes, including eyelid contour, weight prominence, weight migration, improvement of lagophthalmos, and eyelid ptosis, between the two groups were performed using the generalized estimating equation model adjusted for correlation between observations of outcomes from the same patient data measured by five surgeons. The primary outcomes for each grader surgeon data were compared using a binary regression model. Relative risks (risk ratios [RRs]) were performed to express the effect size of the exposure group compared with the standard group on outcomes related to the cohort study design. Statistical significance was set at *p* < 0.05. All data were analyzed using Stata 14.0 (StataCorp. 2015).

## Results

**Fig. 4:**
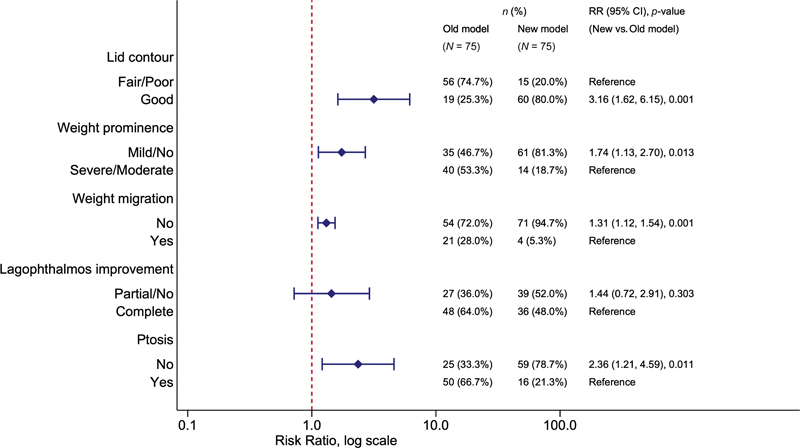
Comparisons of the outcomes of both groups using a generalized estimating equation model adjusted for correlation between observations from same patient (five observations each).

The medical records of the 30 patients included in this study were reviewed. Fifteen patients underwent implantation of commercially available rectangular pretarsal weights (PT group), whereas 15 patients underwent implantation of the newly designed elliptical supratarsal weights (ST group). The average age of the patients was 60.8 ± 12 years, and the majority were female (76%). The average duration of follow-up after surgery was 28 months (range, 25–42 months). Table 1 shows the baseline characteristics of the patients. All patients in the PT group underwent primary implantation, whereas 7 of the 15 patients in the ST group underwent revision surgery to correct weight exposure (4/7) and weight-induced ptosis (3/7). The causes of facial palsy were surgically induced (27 patients), post-traumatic defects (2 patients), and encephalitis (1 patient). Fig. 4 shows the 12-month clinical outcomes of the PT and ST groups. The ST group had significantly better eyelid contour (RR 3.16; 95% confidence interval [CI] 1.62–6.15; *p* = 0.001) than the PT group. The PT group had a significantly higher rate of weight prominence (RR 1.74; 95% CI 1.13–2.7; *p* = 0.013), weight migration (RR 1.31; 95% CI 1.12–1.54; *p* = 0.001), and weight-induced eyelid ptosis (RR 2.36; 95% CI 1.21–4.59; *p* = 0.011) than the ST group. There was no statistically significant difference between the two groups in improvement of lagophthalmos (RR 1.44; 95% CI 0.72–2.91; *p* = 0.303). The reoperative rate at 24 months was significantly higher in the PT group compared with the ST group (53.3 vs. 13.3%; RR 2.00; 95% CI 1.15–3.49; *p* = 0.015). In PT group, reoperation was due to weight exposure (4/8), weight-induced ptosis (3/8), and poor eyelid contour (1/8). In ST group, reoperation was due to weight migration (2/15).

**Table 1:** Baseline characteristics of the patients

Characteristics	Traditional rectangular pretarsal implantation; PT group ( *N* = 15)	Newly designed elliptical supratarsal implantation; ST group ( *N* = 15)
Sex: female	12 (80.0%)	11 (73.3%)
Age (years), mean ± SD	60.7 ± 12.9	60.8 ± 11.0
Operated eye: right eye	10 (66.6%)	8 (53.3%)
Cause of facial palsy		
Surgically induced	14 (93.3%)	13 (86.7%)
Infection	0 (0.0%)	1 (6.7%)
Trauma	1 (6.7%)	1 (6.7%)
Duration of follow-up (months) Mean (Q1, Q3)	30 (26, 42)	27 (25, 30)

Abbreviation: SD, standard deviation.

## Discussion

The design of gold weights and their materials, and the development of implantation techniques, have gradually improved to minimize long-term complications. Implantation in the pretarsal space or low placement of the eyelid weight was a standard lid loading procedure; however, visibility and exposure of the implant are common unfavorable outcomes of this technique. 3 4 11 With time, pretarsal weights become more apparent as orbicularis oculi muscle atrophy occurs. Supratarsal implantation or high placement, which was introduced in 2004, has become more popular because it induces less astigmatism and is associated with less implant visibility as a weight is covered by more tissue planes. 8 9 10 12 In 2018, Allen has summarized the treatment options of the paralytic lagophthalmos and concluded that a supratarsal position of the upper lid weight should be considered rather than pretarsal placement. 2 The only disadvantages of supratarsal placement are more complex surgical steps and more difficult weight sizing.

All commercially available implants are designed for pretarsal implantation of a Caucasian eye with a trapezoid tarsal shape; thus, their dimensions are according to tarsal height and width. The traditional weight dimensions are typically 4.5 to 6.6 mm wide, 9.9 to 18.7 mm long, and 0.6 to 1.0 mm thick. With pretarsal placement of the traditional weight, visibility of the edge of the implant is more frequently observed in Asian eye with sickle-shaped tarsus which is rounder and shorter. 7 Considering of the relatively large area beneath the levator aponeurosis, the new gold weight, which has a larger surface area and is thinner than the traditional gold weight, was specifically designed for supratarsal placement in Asian eye.

The ST group in the present study showed significantly better outcomes and had a lower reoperation rate than the PT group. Moreover, the long-term complications recorded in the ST group were comparable or even less than those reported in the previously published data on platinum-based implants. However, the complete correction of the lagophthalmos was less successful in ST group but did not reach statistical significance. In previous studies, patients implanted with a platinum chain and platinum segment had a reoperation rate of 22.2% at 12 months and 29.5% at 9.1 ± 9.2 months after primary implantation, respectively. 13 14 Whereas the ST group in the present study had a reoperation rate of 13.3% at 24 months. It is universally accepted that platinum is a preferable material to gold because it causes no allergic reactions and has a higher density. However, the limitations of platinum are its higher cost and the complexity of its manufacturing process, which hinders its use in many regions of the world. Platinum chains and segments are less prominent and maintain good eyelid contour compared with platinum plates due to their molding capability and weight distribution across the tarsus. 13 14 15 16 The study also explored the supratarsal postseptal placement of a platinum plate in Asian eyes, which exhibited a low reoperative rate and yielded favorable aesthetic outcomes. 17 Additional research is warranted to investigate the production of a new model employing platinum and its placement beneath the levator aponeurosis.

This study has a few limitations. First, this study was conducted on East Asians, whose eyelid anatomy is different from those of Caucasians and Hispanics. Modification of the supratarsal weight may be needed when using the implants in patients of other ethnicities. However, Caucasians and Hispanics possess a wider superior tarsal border and post-levator space, which should theoretically be compatible with the supratarsal model. Second, the number of cases reviewed in this study was too small to determine the complications associated with the implantation of the new supratarsal gold weight. Third, the superior results in ST group were also induced by the supratarsal implantation technique, not solely the implant design. Finally, the retrospective design of this study is another notable limitation. Long-term collation of the prospective data of a larger number of patients implanted with this new weight is ongoing and the surgical outcomes seem promising.

In conclusion, the newly designed elliptical supratarsal gold weight model evaluated in the present study showed good functional and esthetic outcomes with a lower reoperation rate in primary gold weight implantation and revision surgeries.
